# Identification of MicroRNAs and their Targets Associated with Embryo Abortion during Chrysanthemum Cross Breeding via High-Throughput Sequencing

**DOI:** 10.1371/journal.pone.0124371

**Published:** 2015-04-24

**Authors:** Fengjiao Zhang, Wen Dong, Lulu Huang, Aiping Song, Haibin Wang, Weimin Fang, Fadi Chen, Nianjun Teng

**Affiliations:** 1 College of Horticulture, Nanjing Agricultural University, Nanjing, China; 2 Jiangsu Province Engineering Lab for Modern Facility Agriculture Technology & Equipment, Nanjing, China; 3 China Rural Technology Development Center, Beijing, China; Beijing Forestry University, CHINA

## Abstract

**Background:**

MicroRNAs (miRNAs) are important regulators in plant development. They post-transcriptionally regulate gene expression during various biological and metabolic processes by binding to the 3’-untranslated region of target mRNAs to facilitate mRNA degradation or inhibit translation. Chrysanthemum (*Chrysanthemum morifolium*) is one of the most important ornamental flowers with increasing demand each year. However, embryo abortion is the main reason for chrysanthemum cross breeding failure. To date, there have been no experiments examining the expression of miRNAs associated with chrysanthemum embryo development. Therefore, we sequenced three small RNA libraries to identify miRNAs and their functions. Our results will provide molecular insights into chrysanthemum embryo abortion.

**Results:**

Three small RNA libraries were built from normal chrysanthemum ovules at 12 days after pollination (DAP), and normal and abnormal chrysanthemum ovules at 18 DAP. We validated 228 miRNAs with significant changes in expression frequency during embryonic development. Comparative profiling revealed that 69 miRNAs exhibited significant differential expression between normal and abnormal embryos at 18 DAP. In addition, a total of 1037 miRNA target genes were predicted, and their annotations were defined by transcriptome data. Target genes associated with metabolic pathways were most highly represented according to the annotation. Moreover, 52 predicted target genes were identified to be associated with embryonic development, including 31 transcription factors and 21 additional genes. Gene ontology (GO) annotation also revealed that high-ranking miRNA target genes related to cellular processes and metabolic processes were involved in transcription regulation and the embryo developmental process.

**Conclusions:**

The present study generated three miRNA libraries and gained information on miRNAs and their targets in the chrysanthemum embryo. These results enrich the growing database of new miRNAs and lay the foundation for the further understanding of miRNA biological function in the regulation of chrysanthemum embryo abortion.

## Introduction

microRNAs (miRNAs) are 21–24 nucleotide-long primary endogenous small RNAs that regulate gene expression at the translational level [1,2], including DNA elimination, mRNA cleavage, deadenylation and translational repression [3]. Generally, the miRNAs initially identified in plants, which have been found in many organisms, may serve as key signature molecules that regulate transcription factors and other genes in flowering plant development [4,5], such as flowering time, floral organ patterning and ovule development [6,7]. Based on their extensive integration within genetic networks, the mechanism for miRNA biogenesis in plants has been well investigated in *Arabidopsis thaliana* [8–10].

Chrysanthemum (*Chrysanthemum morifolium*) is an internationally recognized floricultural crop, is the second most important ornamental in the world and has a profound effect on flower production. In addition to aesthetic value, the chrysanthemum is used as a vegetable, in tea and medicine [11,12]. At present, hybridization is the most widely used conventional breeding program for producing new garden phenotypes and optimizing biotic and abiotic resistance, yet embryo abortion is ubiquitous and a primary limiting factor in cross breeding [13–15]. It is widely accepted that normal embryonic development is essential for successful reproduction. In fact, a considerable proportion of ovules fail to produce seeds in many species, even after successful fertilization. In the last few decades, the reproductive barrier has received increased attention. Whereas previous reports have primarily focused on morphological, anatomical [14], and physiological aspects [16–18], very limited information is available about the importance of post-transcriptional regulation (and posttranscriptional regulators) during this process [19]. Therefore, high-throughput sequencing of small RNA pools could provide insight.

Recently, great efforts have been made to identify miRNAs, their targets and functions, and large-scale sequencing projects have enriched the information of miRNAs and targets at both the sequence and expression level in different plant species. Meanwhile, significant progress has been made towards understanding the role of miRNAs in plant development. For instance, one study demonstrated that miR166/165 and its target genes regulate shoot apical meristem and floral development [20]. Other research concerning the importance of miRNA metabolism in normal plant development showed that miR396 controls carpel number and pistil development via regulation of the GRF/GIF (*GROWTH-REGULATING FACTOR*/*GRF-INTERACTING FACTOR*) complex [21]. In addition, some studies have clearly shown that constitutive miR159 or miR167 expression caused male sterility by reducing their target gene expression in Arabidopsis [22–24]. In embryo development research [25,26], cellular differentiation [27], embryonic pattern formation and developmental timing [28] have been reported, suggesting that they have important functions in embryonic development. In addition, more than 400 miRNA targets have been identified [29]. However, the crucial functions of most miRNAs that regulate embryo patterning are still unknown [30]. Hence, we explored the relationship between miRNAs and embryo abortion through high-throughput sequencing. As a consequence, we revealed the expression level of numerous key miRNAs and their targets related to embryo abortion, which expands our understanding of the miRNA pathway in embryonic development.

## Methods

### Plant materials

*C*. *morifolium* ‘Yuhualuoying’ (2n = 6X = 54) is a ground-cover chrysanthemum cultivar with highly excellent ornamental traits and important commercial quality, but weak resistance to several abiotic factors. *C*. *nankingense* (2n = 2X = 18) is a wild species with strong adaptability, such as cold tolerance, and it is an important parent in chrysanthemum cross breeding. However, distant hybridization between cultivar and wild species is difficult. In this hybrid combination, the offspring failed to generate seeds and found that the vast majority of embryos aborted at 18 DAP. Here, ‘Yuhualuoying’ and *C*. *nankingense* were used as the female and male parents, respectively. They were maintained in the Chrysanthemum Germplasm Resource Preserving Centre, Nanjing Agricultural University, China (32°05’ N, 118°90’ E). Interspecific crosses were performed according to previously described methods [13]. Sample collection was performed as described previously [31]. Briefly, 60 chrysanthemum plants were planted in three planting areas, with 20 plants in each area. At 12 days after pollination, when nearly all the embryos were normal and reached the globular phase, approximately 700 normal ovules were collected (approximately 0.2 g) from each planting area, and a total of more than 0.6 g ovules were collected at this stage. At 18 days after pollination, when normal embryos grew into heart-shaped embryos and abnormal embryos began degeneration, >0.6 g normal and abnormal ovules were collected. After collection, the three samples, normal embryos at 12 DAP (NE12), normal embryos at 18 DAP (NE18) and abortive embryos at 18 DAP (AE18), were immediately frozen in liquid nitrogen and stored at -80°C.

### Total RNA preparation and small RNA library construction

Total RNA was extracted from three samples with Trizol reagent according to the manufacturer’s protocol (Takara Bio Inc., Otsu, Japan). The integrity and purity of the total RNA was determined by an Agilent 2100 RNA 6000 Kit and electrophoresis on a 1% agarose gel. RNAs were stored at -80°C until being used for small RNA sequencing.

### Small RNA library construction and high-throughput sequencing

The samples were subjected to 15% denaturing polyacrylamide gel electrophoresis and small RNAs with 18–30 nt fragments were purified. The small RNA molecules were sequentially ligated to 5’ and 3’ adaptors and were reversed transcribed into DNA by RT-PCR. Finally, three RT-PCR products were sequenced by the Beijing Genomics Institute (BGI) (Shenzhen, Guangdong Province, China) using the Solexa sequencing method, and three small RNA libraries were constructed. The deep-sequencing dataset has been deposited in NCBI, and the accession number is PRJNA268153.

### Bioinformatics analysis of sequencing data

The raw sequences, which are not always effective, were processed as described by Sunkar et al. [32]. The modified 18–30 nt sequences were obtained from the raw sequences by removing the adaptor sequences for further analyses. Firstly, rRNA, tRNA, snRNA, snoRNA and those containing a poly-A tail were omitted from the sRNA sequences, and then the remaining sequences were compared against the NCBI Genbank database and Rfam 11.0 [33] databases. To identify conserved miRNAs in chrysanthemum embryos, the unique sRNA sequences were compared with the miRBase 19.0 [34] using a BlastN search. Only perfectly matched sequences were considered to be conserved miRNAs.

### Differential miRNA expression analysis related to embryo development

miRNA expression among the three samples was compared to identify differences in miRNA expression related to embryonic development, as follows: miRNA expression was normalized in the three samples (NE12, NE18 and AE18) to obtain the expression of transcript per million (TPM). Normalized expression = Actual miRNA count/Total count of clean reads*1000000. Notably, if the miRNA gene expression was zero after normalization in any one of the two samples, then it was revised to 0.01; if the miRNA gene expression of the two samples was less than 1, it was not used in the analysis of differential expression because of low expression. The fold change between two samples was calculated as: fold change = log_2_ (sample 1/sample 2) [35]. To clearly display the expression profile, a heatmap was generated using Cluster v3.0 software and visualized using Treeview [36].

### Prediction of potential chrysanthemum miRNA target mRNAs

To understand the molecular function of differentially expressed miRNAs in chrysanthemum embryos, miRNAs were analyzed using TargetScan [37] to predict their target mRNAs. Newly identified chrysanthemum miRNA sequences were used as custom miRNA sequences, and the chrysanthemum transcript library built from these was used as a custom plant database. A total of 615,312 contigs and 116,697 unigenes were identified, and all of them had the Nr, Nt, COG, GO and KEGG annotation. More details have been previously described in an article about chrysanthemum embryo transcriptomic analysis [31]. Genes were classified according to KEGG functional annotations using the DAVID bioinformatics resources [38]. Moreover, GO term enrichment of the target genes was calculated with the use of Gene Ontology (GO) project (http://www.geneontology.org/).

### Validation of miRNAs through real-time quantitative PCR (qRT-PCR)

To validate the high-throughput sequencing results of chrysanthemum embryo miRNAs, 21 differentially expressed miRNAs were randomly selected and analyzed by qRT-PCR. RNA samples were reverse-transcribed using PrimeScript miRNA qPCR starter kit ver 2.0 (Takara Bio). A final volume of 20 μL was achieved by the addition of 5 pmol of the forward and the reverse primers (S1 Table). The conditions for the PCR amplification were as follows: polymerase activation at 95°C for 3 min, followed by 40 cycles of 95°C for 20 s, 60°C for 20 s and 72°C for 45 s. All reactions were performed in triplicate on a Mastercycler ep realplex device (Eppendorf, Hamburg, Germany) according the kit’s protocol. The *EF1α* gene was used as the reference gene [39], and relative expression levels were calculated by the 2^-△△CT^ method [40].

### Target validation by 5’ RNA Ligase-Mediated Rapid Amplification of cDNA Ends (RLM-RACE)

To validate the predicted target cleavage sites, a modified procedure for RLM-RACE [41] was performed using the FirstChoice RLM-RACE Kit (Ambion, Austin, TX, USA). This protocol started with 2 μg mixed total RNA from the three embryo samples. All reactions were performed using following steps: (i) 5’ RACE adapter ligation. The components of reaction included 2 μL total RNA (1000 ng/μL), 1 μL 5’ RACE adapter, 1 μL 10X RNA ligase buffer, 2 μL T4 RNA ligase (2.5 U/μL) and 4 μL nuclease-free water, which were gently mixed, briefly spun, and incubated at 37°C for one hour. (ii) Reverse transcription (RT). All reactions were carried out in a total volume of 20 μL, including 2 μL ligated RNA, 4 μL dNTP mix, 2 μL random decamers, 2 μL 10X RT buffer, 1 μL RNase inhibitor, and 1 μL M-MLV reverse transcriptase. After mixing and spinning, the reactions were incubated at 42°C for one hour. (iii) Nested PCR for 5’ RLM-RACE. 5’ RACE outer primer and gene-specific outer primer were used for outer 5’ RLM-RACE. A total of 1 μL RT reaction mixed with 2.5 μL 10X PCR buffer, 2 μL dNTP mix, 1 μL 5’ RACE gene-specific outer primer (10 μM) and 1 μL 5’ RACE outer primer were added to a final volume of 25 μL. The conditions were as follows: initial denaturation at 94°C for 3 min, followed by 35 cycles of 94°C for 30 s, 60°C for 30 s, 72°C for 1 min, with a final extension at 72°C for 7 min. Nested PCR was performed using 1 μL outer PCR product, the 5’ RACE inner primer, gene-specific inner primer and other components. The conditions were same as above for outer 5’ RLM-RACE. The 5’ RACE primer and gene-specific primers are shown in S2 Table. After nested PCR, the 5’ RACE products were purified using the Agarose Gel DNA Purification Kit (TaKaRa Bio), ligated into the pMD19-T vector (TaKaRa Bio), and sequenced [42].

## Results

### Construction of small RNA libraries by high-throughput sequencing

**Fig 1:**
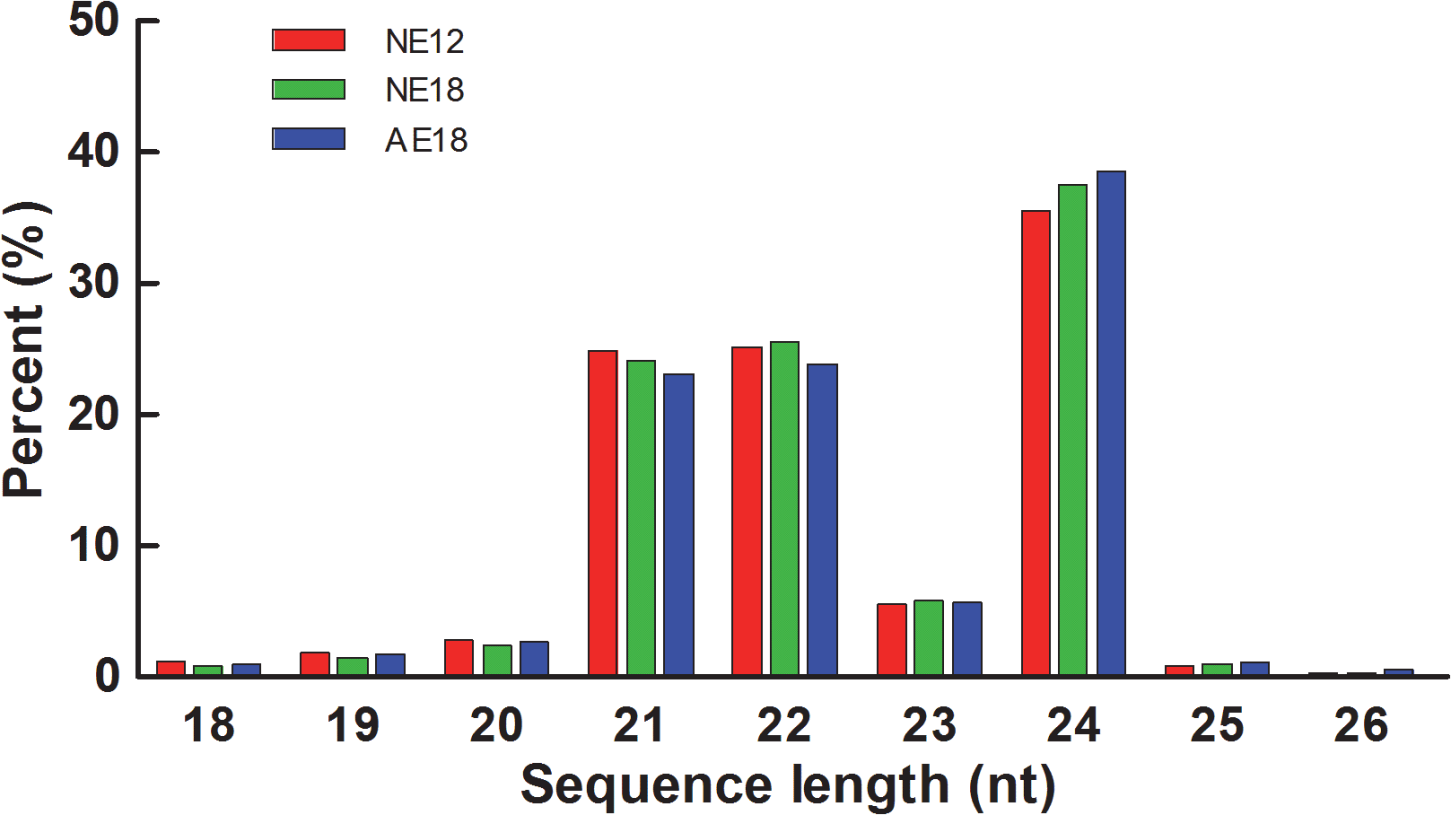
Lengths of unique small RNA sequences in chrysanthemum.

To identify miRNAs involved in chrysanthemum embryo development, we first used the high-throughput sequencing technique to construct three libraries from NE12, NE18 and AE18. A total of 10526337, 11922890 and 10663240 reads were obtained from the three libraries, respectively (Table 1), after filtering out reads without small RNA sequences. The sequences ranged from 18 to 30 nt in length (Fig 1), of which the majority were 19 to 25 nt long, and 24 nt small RNAs were most highly enriched in the three libraries. We next obtained clean reads after adaptor sequence removal, low quality tags, contaminants and short RNAs <18 nt, i.e., rRNA, snRNA, snoRNA, tRNA and unannotated reads (Table 2).

**Table 1:** Summary of filtered data produced by small RNA sequencing.

Type	NE12		NE18		AE18	
	Count	Percent (%)	Count	Percent (%)	Count	Percent (%)
Total reads	10526337		11922890		10663240	
High quality	10406754	1	11797964	1	10544367	1
3' adapter null	17919	0.17	19688	0.17	20656	0.2
Insert null	863	0.01	1454	0.01	806	0.01
5' adapter contaminants	6565	0.06	5903	0.05	5982	0.06
Smaller than 18 nt	195505	1.88	118586	1.01	128648	1.22
PolyA	484	0	1136	0.01	937	0.01
Clean reads	10185418	97.87	11651197	98.76	10387338	98.51

**Table 2:** Distribution of small RNAs among different categories in chrysanthemum.

	NE12				NE18				AE18			
Category	Unique sRNAs	Percent(%)	Total sRNAs	Percent(%)	Unique sRNAs	Percent(%)	Total sRNAs	Percent(%)	Unique sRNAs	Percent(%)	Total sRNAs	Percent(%)
**Total**	3266988	100	10185418	100	3741798	100	11651197	100	3403142	100	10387338	100
**miRNA**	10117	0.31	327244	3.21	9436	0.25	267683	2.3	8714	0.26	261684	2.52
**rRNA**	43812	1.34	322625	3.17	59827	1.6	443510	3.81	57728	1.7	402156	3.87
**snRNA**	935	0.03	1747	0.02	1139	0.03	2312	0.02	1146	0.03	2013	0.02
**snoRNA**	232	0.01	352	0	375	0.01	639	0.01	337	0.01	555	0.01
**tRNA**	7942	0.24	78453	0.77	9451	0.25	84273	0.72	12382	0.36	118948	1.15
**Unannotated**	3203950	98.07	9454997	92.83	3661570	97.86	10852780	93.15	3322835	97.64	9601982	92.44

Deep sequencing results of the three libraries showed that the majority of small RNAs were 21–24 nt long, in agreement with the typical range for plant miRNAs, and 24 nt small RNAs were the most abundant. The abnormal 18 DAP embryos had the highest expression levels (Fig 1). However, the distribution of each category of small RNA was distinct in each of the three libraries. For example, NE12 expressed the highest number of miRNAs, while AE18 expressed the least. The proportion of snRNAs and tRNAs in AE18 was higher than the other two libraries (Table 2).

### Identification of known miRNAs

To identify miRNA expression abundance in the chrysanthemum embryo, we compared the sRNA library to known plant miRNAs in miRBase 19.0 by BlastN. After sequence analysis allowing one or two mismatches between sequences, we identified 228 miRNAs in the three RNA libraries (S1 Dataset). By analyzing the number of these miRNA reads, we found that there were significant changes in their expression frequency.

### Differentially expressed miRNAs between normal and abnormal embryos

**Fig 2:**
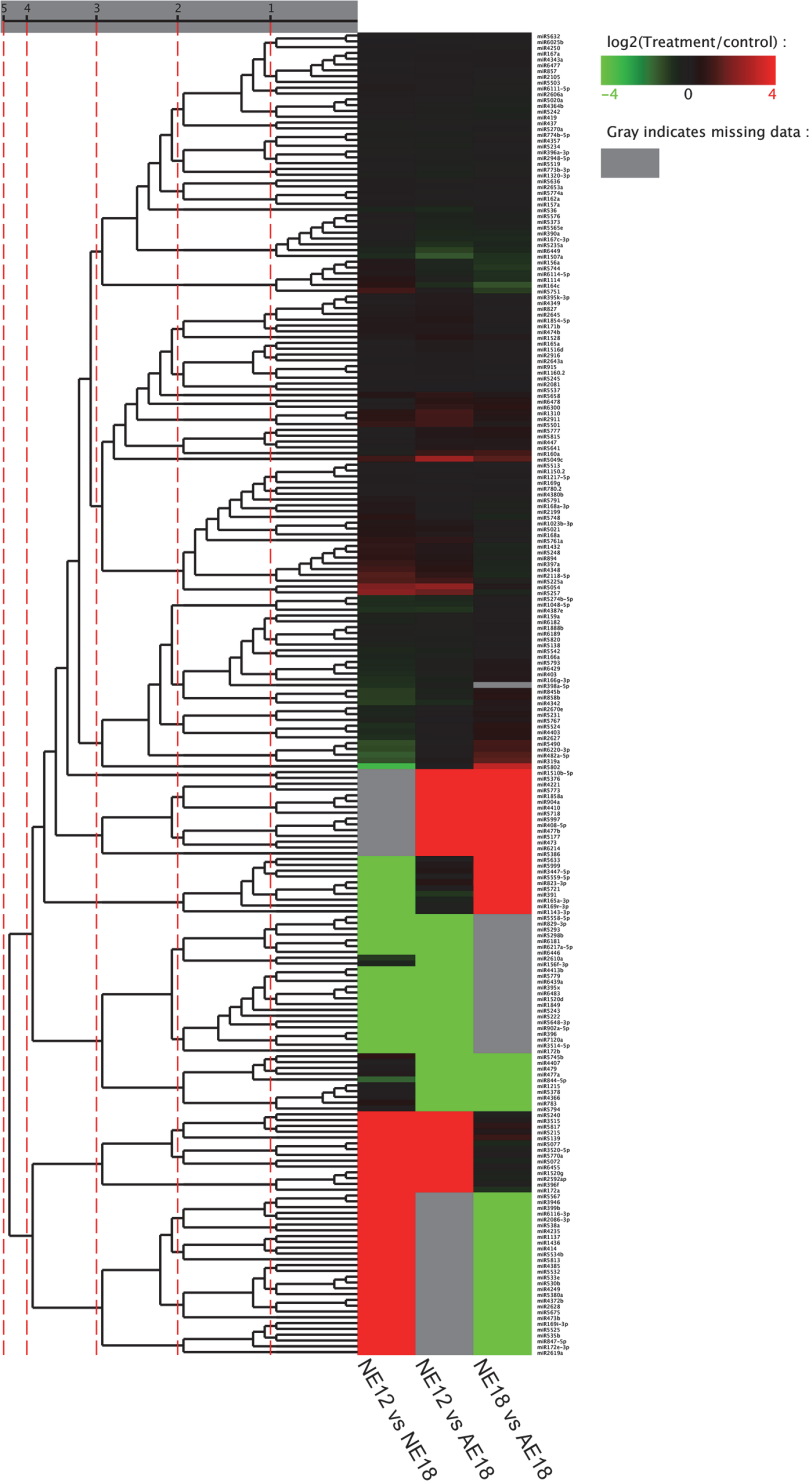
Heatmap of differential expression analysis and cluster analysis of 228 known miRNAs between samples. The bar represents the scale of the expression abundance for each miRNA (log_2_ TPM of treatment/control) in three comparable groups as indicated by the red/green rectangles. Red rectangles represent up-regulation and green rectangles represent down-regulation. All information for each miRNA can be found in S1 Dataset.

The outcome of sequence counts indicated that some miRNAs have relatively more counts, suggesting these miRNAs are preferentially expressed throughout embryonic development. For instance, miR166a, miR157a and miR156a had more than 7000 counts in the three libraries. In contrast, several other miRNAs showed specific expression in a subset of the libraries. For example, miR172b was only expressed in normal embryos (NE12), as its expression in NE18 and AE18 was zero, and miR1143-3p was highly expressed in NE12 and AE18, but had low expression in NE18. To understand the expression of these known miRNAs, we employed differential expression analysis and cluster analysis of the 228 known miRNAs, and the results are listed in one heatmap (Fig 2).

To explore the specific miRNAs related to embryo development, we compared the normalized expression of miRNAs in the three small RNA libraries pairwise. All differentially expressed miRNAs with *p*-values less than 0.05 and expression levels greater than 2-fold-change are shown in S2 Dataset. We found that there were 85, 72 and 69 differentially expressed miRNAs in the following three comparisons: (a) NE12 vs. NE18; (b) NE12 vs. AE18; (c) NE18 vs. AE18, respectively (S2 Dataset).

### Target prediction for known miRNAs

**Fig 3:**
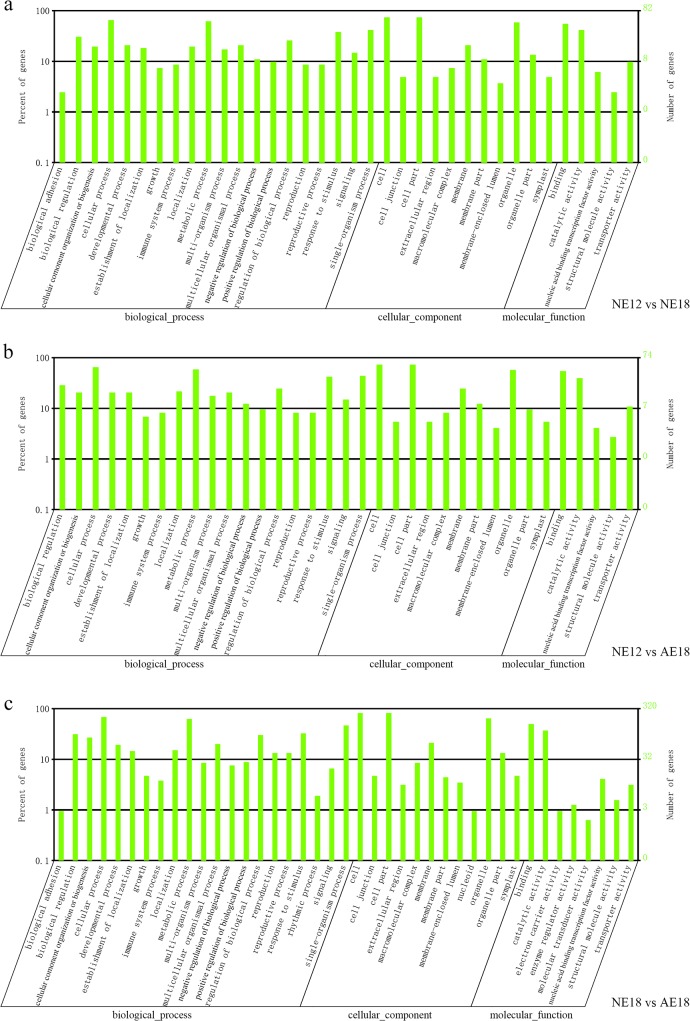
Gene Ontology (GO) classifications of putative targets in the chrysanthemum embryo.

To understand the biological function of these miRNAs, it is vital to identify their potential targets. We predicted a total of 1037 potential target genes for 129 identified miRNAs from the transcripts of chrysanthemum embryo libraries (Sheet 1 in S3 Dataset). All annotated information of these targets is provided (Sheet 2 in S3 Dataset). According to the Blastx results, sequences corresponding to the putative targets were associated with three ontologies in Gene Ontology (GO): molecular functions, cellular components and biological processes (Fig 3). In the three above-mentioned comparisons, the top four GO terms were cell, cell part, cellular process and metabolic process. On the contrary, few transcripts were annotated to genes involved in structural molecule activity.

In addition, miRNA target genes were related to transcription factors and embryo development. As a result, we identified 52 predicted target genes related to embryo development, including 31 transcription factors and 21 other genes (Tables 3 & 4). We found that the majority of the transcription factors are known, such as auxin response factor (ARF) family (regulated by miR160, 167, 414), which had the highest number, homeobox leucine-zipper protein (targeted by miR165 and miR166), MYB family (targeted by miR159, 319, 414, 1114, 5298, 5658), ethylene-responsive transcription factor (targeted by miR172), and WRKY DNA binding protein (regulated by miR5380) (Table 3). Moreover, GO enrichment analysis provided the main biological functions of the target genes. The results indicated that 21 genes participated in various reproductive processes, including embryonic pattern specification (GO:0009880), embryo development (GO:0009790), post-embryonic development (GO:0009791) and embryo development ending in seed dormancy (GO:0009793) (Table 4). In short, our prediction of potential targets indicated that these genes are involved in a wide range of plant biological processes during chrysanthemum embryo development.

**Table 3:** Identified candidate transcription factor targets of miRNAs during chrysanthemum embryogenesis.

miRNA	Target gene ID	NE2_FPKM	NE18_FPKM	AE18_FPKM	annotation
miR159	CL9206.Contig1_All	8.8268	6.6441	5.7653	GAMYB-like2
miR160	CL6013.Contig2_All	25.4787	21.3715	17.7767	auxin response factor 18
	Unigene17545_All	26.0161	21.019	19.025	auxin response factor 10
	Unigene374_All	12.8624	8.336	8.1864	auxin response factor
	Unigene7539_All	7.7804	11.3549	13.5256	auxin response factor 16
miR165	CL2494.Contig1_All	17.2011	14.7895	13.3594	homeobox leucine-zipper protein
miR166	CL1275.Contig3_All	13.1222	6.1693	4.7337	homeobox leucine-zipper protein
	CL2494.Contig1_All	17.2011	14.7895	13.3594	homeobox leucine-zipper protein
miR167	CL1486.Contig3_All	1.7527	1.5281	1.474	auxin response factor 1
	Unigene13456_All	38.8213	26.3134	30.6115	auxin response factor 6
	Unigene3472_All	0.6448	0.3355	0.2665	auxin response factor 1
	Unigene3474_All	5.8209	3.5086	3.3267	auxin response factor 1
miR172	CL12144.Contig2_All	3.6013	2.0811	2.824	ethylene-responsive transcription factor RAP2-7-like
miR319	CL9206.Contig1_All	8.8268	6.6441	5.7653	GAMYB-like2
miR414	CL11642.Contig1_All	89.5743	81.5577	83.1779	Zinc finger protein
	CL2007.Contig4_All	2.8762	4.4129	3.3445	calmodulin-binding transcription factor SR2L
	CL7290.Contig1_All	16.3852	14.6688	16.6145	ATERF3/ERF3
	Unigene1442_All	6.7029	13.6356	10.7171	SAC51 transcription factor
	Unigene5061_All	162.267	100.757	94.5308	MADS-box transcription factor CDM104
	Unigene5423_All	45.6283	54.7193	56.8421	bHLH122
	Unigene630_All	49.7407	100.5877	133.5406	auxin response factor 19
	Unigene8542_All	4.5087	3.9645	4.4534	bHLH110-like
	Unigene9425_All	2.1801	1.0677	1.2358	Myb-like protein A
miR894	CL7400.Contig1_All	3.0342	2.1561	2.7542	WUSCHEL-related homeobox 13-like
miR1114	Unigene17113_All	2.9068	5.5519	3.9821	MYB2
	Unigene47172_All	3.9374	14.8477	13.206	MYB68
miR2606	CL13855.Contig12_All	1.8751	0.7562	1.0421	APETALA2
miR5298	CL4971.Contig2_All	5.7086	2.8565	3.9865	Myb family protein-like protein
miR5380	Unigene12888_All	0	0.2626	0.1689	WRKY DNA binding protein
	Unigene29088_All	0	0.087	0	WRKY DNA binding protein
miR5658	CL4971.Contig2_All	5.7086	2.8565	3.9865	Myb family protein-like protein

**Table 4:** Identified candidate miRNA targets associated with embryo development.

miRNA	Target gene ID	NE12_FPKM	NE18_FPKM	AE18_FPKM	annotation	GO-Biological Process
miR168	Unigene26402_All	50.0516	37.365	33.2644	AGO1-1	GO:0009880// embryonic pattern specification
	Unigene33573_All	13.3169	9.6999	9.4405	AGO1-1	GO:0009880// embryonic pattern specification
miR396	CL15279.Contig11_All	13.8518	9.3287	8.9313	RNA-dependent RNA polymerase 5	GO:0009793// embryo development ending in seed dormancy
miR414	CL13418.Contig10_All	4.7248	8.388	6.5565	DNA polymerase epsilon catalytic subunit A	GO:0009793// embryo development ending in seed dormancy
	CL4347.Contig1_All	5.7647	3.5528	2.3867	26S proteasome non-ATPase regulatory subunit 4-like isoform 2	GO:0048569// post-embryonic organ development
	CL4558.Contig1_All	7.635	5.4736	6.0619	thiosulfate sulfertransferase	GO:0009793// embryo development ending in seed dormancy
	CL5512.Contig1_All	2.4496	2.5036	1.8112	DNA replication licensing factor mcm2	GO:0009790// embryo development
	Unigene1949_All	50.2206	38.0872	36.5404	RNA-binding protein, contains THUMP domain	GO:0009793// embryo development ending in seed dormancy; GO:0009560// embryo sac egg cell differentiation
	Unigene6954_All	11.1434	6.1588	6.4912	histone arginine demethylase JMJD6	GO:0009793// embryo development ending in seed dormancy
	Unigene8527_All	29.4372	20.146	24.2973	Chloroplastic group IIA intron splicing facilitator CRS1	GO:0009793// embryo development ending in seed dormancy
miR1114	Unigene3202_All	4.9467	2.4764	3.8427	Separin	GO:0009793// embryo development ending in seed dormancy
miR5234	Unigene16643_All	5.8117	4.0253	3.6867	protein embryo defective 1703	GO:0009793// embryo development ending in seed dormancy
miR5567	Unigene11829_All	2.0712	1.9773	1.8577	ARID and Hsp20 domains containing protein	GO:0009560// embryo sac egg cell differentiation
miR5576	Unigene8468_All	12.2968	6.6835	6.7827	EMB2410	GO:0009793// embryo development ending in seed dormancy
miR5641	CL1036.Contig2_All	0.8155	0.4992	0.5136	splicing factor 3A subunit 3-like	GO:0009793// embryo development ending in seed dormancy
	Unigene10751_All	8.0763	5.0022	6.3961	arginyl-tRNA synthetase-like	GO:0009793// embryo development ending in seed dormancy
miR5813	Unigene23217_All	16.1426	13.3944	15.5322	Helicase	GO:0009793// embryo development ending in seed dormancy
miR6111-5p	Unigene14608_All	3.4383	3.1905	3.8469	receptor-like kinase	GO:0009791// post-embryonic development
miR6182	CL14437.Contig1_All	1.2063	0.9108	1.1397	flowering time control protein FCA	GO:0009791// post-embryonic development; GO:0009790// embryo development
miR6189	Unigene20704_All	23.6094	22.8058	21.6907	protein phosphatase 1 regulatory subunit 11-like	GO:0009790// embryo development
miR6439	CL7205.Contig2_All	14.7975	12.0563	11.9233	14-3-3 family protein	GO:0009791// post-embryonic development

### qRT-PCR validation of miRNA expression level

**Fig 4:**
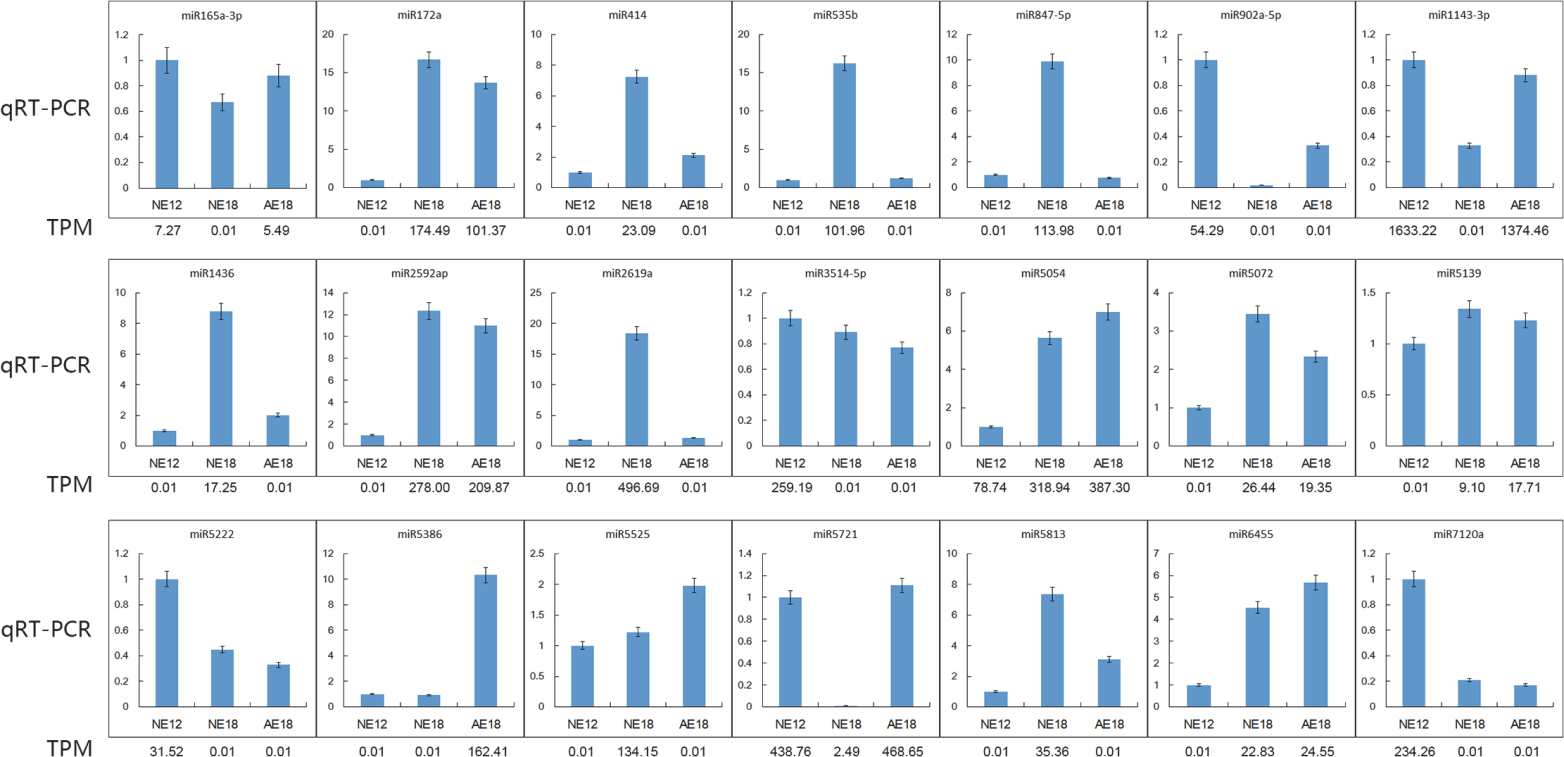
Validation of 21 known miRNAs by qRT-PCR. TPM is their abundance in the sequencing data of the sRNA libraries.

To validate the dynamic miRNA expression pattern identified by deep sequencing, we randomly selected 21 predicted candidate miRNAs from the three libraries and validated them by qRT-PCR. The expression patterns of the 21 miRNAs are shown in Fig 4, which had different expression patterns in the normal and abortive embryo. These miRNAs include miR414, miR847-5p, miR1436, miR2619a, and miR5813, all of which had a similarly high expression pattern in the normal embryo at 18 days. In comparison, the expression levels of miR1143-3p, miR9025-5p and miR5386 were higher in AE18 compared to NE18. Other miRNAs showed differential expression at different developmental stages. We compared the expression level of each miRNA in the three samples to its abundance (TPM) in the sequencing data of the three libraries. The results indicate that the qRT-PCR results of most miRNAs were consistent with the sequencing data (Fig 4).

### RLM-RACE validation of target prediction

**Fig 5:**
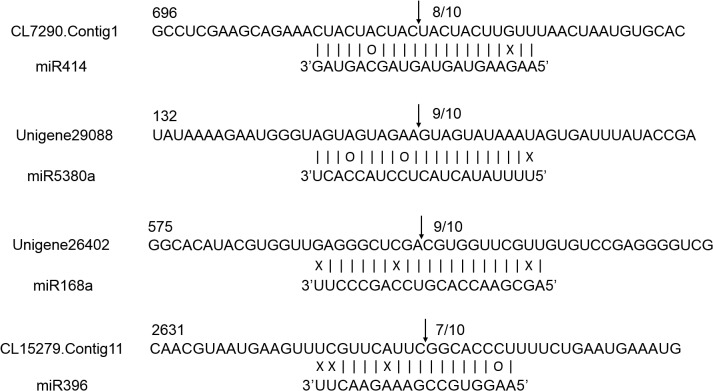
Mapping of mRNA cleavage sites confirmed by 5’ RLM-RACE. Arrows indicate the 5’ ends of mRNA fragments, as identified by cloned 5’ RLM-RACE products, with the frequency of clones shown.

In this study, we used 5’ RLM-RACE to map the cleavage sites in four predicted target genes in chrysanthemum. CL7290.Contig1, Unigene29088, Unigene26402, and CL15279.Contig11 were confirmed as real targets of miR414, miR5380a, miR168a, and miR396, respectively, as all of the 5’ ends of the mRNA fragments mapped to the nucleotide that paired to the tenth nucleotide of each miRNA with higher frequencies than depicted for each pairing oligo (Fig 5).

## Discussion

### Identification of chrysanthemum embryo small RNAs by high-throughput sequencing

miRNAs play essential roles in regulating plant growth and development. In recent years, high-throughput sequencing has provided an efficient method to quantitatively profile small RNA populations. Most conserved and novel miRNAs in plants have been identified, and the plant miRNA database that includes model plants and major crops has been established [43]. To our knowledge, miRNAs have been studied in field of plant reproductive biology. It has previously been suggested that miRNAs are key regulators of the embryonic maturation program, and they regulate the timing of embryogenesis in Arabidopsis [29]. The aim of this work was to identify specific miRNAs by high-throughput sequencing from normal and abnormal chrysanthemum embryos, and to analyze the differentially expressed miRNAs associated with embryo abortion. Here, we report that 24 nt sRNAs were most abundant in the chrysanthemum embryo library, which is consistent with those of *A*. *thaliana* [44], *Oryza sativa* [45], *Medicago truncatula* [46] and *Prunus mume* [35]. Other studies have found that 21nt sRNAs are the most abundant in *A*. *thaliana* [47] and other species [48,49]. These indicate that the small RNA transcriptome is complicated and significantly different in various plant species and organs.

### Identified miRNAs potentially regulate chrysanthemum embryo abortion

It is now clear that miRNA plays a variety of developmental roles during the plant life-cycle. Previous studies have demonstrated that miRNAs regulate the timing of embryo maturation in Arabidopsis [50], regulate hormone biosynthesis and morphogenesis [51]. In this study, we identified 228 known miRNAs from the chrysanthemum embryo by high-throughput sequencing, 129 of which provided information of target prediction from the chrysanthemum embryo transcriptome [31]. Recently, multiple studies have shown that many conserved miRNAs, such as miR156, miR159, miR166, miR167a, miR169, miR390, miR396, and miR397, play regulatory roles during embryonic development [50,52,53]. In a study of *A*. *thaliana* embryonic tissue, the expression pattern of miR396 displayed uniform accumulation in longitudinal sections of the mature green-stage embryo [54]. Here, we found that the miR396 only appeared in normal embryos at 12 days after pollination, when most embryos had reached the globular stage, suggesting that miR396 is related to early embryonic developmental stages. Moreover, we found that miR169r-3p, miR172a, miR172b, miR414, miR482a-5p, miR535b, miR1143-3p, miR2118-5p, miR5054, miR5721, and miR7120a were differentially expressed at different stages of embryo development. In a similar study of *Raphanus sativus* [55], miR169r-3p and miR535b were detected only in the library of 4–15 day after pollinated ovules, while they were not expressed in 0 day after pollinated ovules. As shown, miR169r-3p was repressed in NE18, but it was expressed in AE18. In contrast, miR414 and miR535b displayed the opposite expression pattern, as they were more highly expressed in NE18. The miRNAs with these changes likely have targets that play critical roles in chrysanthemum embryo development, even at the abortion stage. More importantly, we identified some novel and unconserved miRNAs, such as miR3520-5p and miR5054, which significantly increased in embryos at 18 DAP, indicating that these miRNAs may be responsible for embryo morphogenesis and maturation.

### Functions of predicted target genes during chrysanthemum embryo development

miRNAs and their potential targets prediction could provide information about the biological processes regulated by miRNAs. Most miRNAs exert their biochemical or biophysical effects through downstream targets. High-throughput sequencing and bioinformatics have provided us reliable and efficient approaches in several plant species [53,56]. In plants, a dominant portion of target transcripts share short highly complementary regions called target sites with their regulatory miRNAs, through which the targets are silenced [57]. Therefore, we performed 5’ RLM-RACE to validate miRNA-target interaction. Four predicted targets had specific cleavage sites corresponding to the miRNA complementary sequences (Fig 5) and may be regulated by these miRNAs such that the miRNA directs the cleavage of targets [58].

Target genes involved metabolic pathways were highly represented. For example, CL13855.Contig2_All (GO:0044237//cellular metabolic process) had the least expression in AE18, about half the expression in NE12. It has been shown that genes with metabolic activity were expressed during Arabidopsis embryo development [59]. Furthermore, our previous report illustrated that most genes and proteins associated with energy metabolism were significantly down-regulated in AE18 [31]. These predicted target genes displayed temporal expression patterns in normal and abnormal embryos, further confirming the importance of energy metabolism during embryo development.

In *A*. *thaliana*, the phytohormone auxin is an important patterning agent during embryogenesis and post-embryonic development, and it exerts its effects through transcriptional regulation [60,61]. BODENLOS (BDL) and its interacting ARF partner ARF5 play a pivotal role during the earliest stages of embryonic development [62,63], while the HOMEODOMAIN-LEUCINE ZIPPER (HD-ZIP) transcription factor HB5 negatively regulates *BDL* expression. In our results, auxin response factor (ARF) 1, 6, 10, 16, 18, 19 and homeobox leucine-zipper protein were putative miRNA targets during chrysanthemum embryogenesis (Table 3), which suggest a regulation model in which miRNAs may participate in the crosstalk between these factors. In addition, homozygous *monopteros* mutants with mutations in an ARF gene show defects in the development of the basal region of the embryo, leading to seedlings that sometimes possess only a single cotyledon after germination [62]. Our results show that ARF1 expression (Unigene3472_All, Unigene3474_All and CL1486.Contig3_All) was highest in NE12, but lowest in AE18. However, there was no significant difference between other ARFs, suggesting that the ARF1 may play a common role in auxin response in early embryonic development. Other transcription factor families also play important roles in embryonic development, including ethylene-responsive transcription factor [64], MYB [65], WRKY [66] and MADS-box [67,68] transcription factor. We identified the members of these transcription factor families as candidate miRNA targets during chrysanthemum embryogenesis.

In addition to transcription factors, other target genes also participate in embryo development. Previous reports have shown that the argonaute protein family is a key player in small RNA-guided gene-silencing pathways [69]. In Arabidopsis, the ARGONAUTE1 (AGO1) protein acts in the miRNA pathway and is broadly expressed in development [70], while ago1 loss-of-function mutants display pleiotropic defects in development and virus defense [71]. In Drosophila, argonaute-2 (ago-2) is required for proper nuclear migration, pole cell formation, and cellularization during early embryonic development [72]. As shown here, *miR168* was more highly expressed in NE18 than NE12, however, its target gene *AGO1* was more highly expressed in NE12, suggesting that *miR168* may have regulatory roles in embryo development.

## Conclusions

In conclusion, small RNA sequencing and the discovery of different microRNAs provide an extensive perspective into chrysanthemum embryo abortion. Sequence analyses and qRT-PCR validation revealed that some miRNAs have distinct expression levels at different stages of embryo development and are potentially significant to embryo abortion. Prediction and annotation of miRNAs-mediated targets provided favorable information for the future study of gene function, which will provide new insights into the mechanism of embryo development and offer new information about factors that regulate embryo abortion in plants. Moreover, GO categorization and enrichment analysis of differentially expressed genes demonstrate that several transcripts are required for chrysanthemum embryo development. Taken together, our investigation provides valuable information for further functional characterization of miRNAs related to plant embryo development.

## Supporting Information

S1 Dataset Known miRNAs in three libraries. (XLS)

S2 Dataset Differentially expressed miRNAs. (XLS)

S3 Dataset Identified miRNAs and their potential target. (XLS)

S1 Table Primers used for qRT-PCR. (PDF)

S2 Table 5' RACE PCR primer sequences used. (PDF)
