# Supplementary material for: Identification of MicroRNAs and their Targets Associated with Embryo Abortion during Chrysanthemum Cross Breeding via High-Throughput Sequencing
Source: PLoS One. 2015 Apr 24;10(4):e0124371. doi: 10.1371/journal.pone.0124371 (PMC4409343; doi:10.1371/journal.pone.0124371)
Supplement: S1 Table — (PDF) [file pone.0124371.s004.pdf]

S1 Table. Primers used for qRT-PCR.

miRNA	Primer sequences
miR165a-3p	TCGGACCAGGCTTCATCC
miR172a	AGAATCTTGATGATGCTGCAT
miR414	TCATCTTCATCATCATCGTCA
miR535b	TGACAACGAGAGAGAGCACGC
miR847-5p	TCTTGATGAAGAGGAATGGAA
miR902a-5p	TATGATGCAGATTCTTCA
miR1143-3p	TTATTTGCCCGAAGGGGACGTCCT
miR1436	ACATTATGGGACGGAGGGAGT
miR2592ap	AGGCTGGTTTAGATGAAGGTA
miR2619a	ACATAGGAGGCTGTTTTGTAT
miR3514-5p	AGGATTCTGTATTAACGGTGGA
miR5054	TCCCCACGGTCGGCGCCA
miR5072	CGATTCCCCAGCGGAGTCGCCA
miR5139	AAACCTGGCTCTGATACCA
miR5222	TTACAGGAGAAGAATGTATGGC
miR5386	CGTCGCTGTCGCGCGCGCTG
miR5525	TGAACCTTGGGAGCGATCTGAA
miR5721	AAAAATGGAGTGAGAAATGGA
miR5813	AAGCAGCGACTCTGGTCATGGA
miR6455	TCAAATAGCATCCTCAACATT
miR7120a	TGTTATATTGTCAGATTGTCA
