# Supplementary material for: Identification of MicroRNAs and their Targets Associated with Embryo Abortion during Chrysanthemum Cross Breeding via High-Throughput Sequencing
Source: PLoS One. 2015 Apr 24;10(4):e0124371. doi: 10.1371/journal.pone.0124371 (PMC4409343; doi:10.1371/journal.pone.0124371)
Supplement: S2 Table — (PDF) [file pone.0124371.s005.pdf]

S2 Table. 5' RACE PCR primer sequences used.

primer name	5′–3′ sequence
5' RACE outer primer	GCTGATGGCGATGAATGAACACTG
CL7290.Contig1_All outer primer	CTGTGACGGGAACGGAGG
Unigene29088_All outer primer	GTAGTCACCACAACTCCGTCATC
Unigene26402_All outer primer	TTTGACCCAGATTAGGGGAGTAG
CL15279.Contig11_All outer primer	AACAGTCACGTCGTCACTCCTATA
5' RACE inner primer	CGCGGATCCGAACACTGCGTTTGCTGGCTTTGATG
CL7290.Contig1_All inner primer	ATAGCCTAAGCACATTCCATCC
Unigene29088_All inner primer	CATTTGATAGTCTTTGAACTTGCTTCT
Unigene26402_All inner primer	GACACCTTTTCCTGGGCGTA
CL15279.Contig11_All inner primer	TCGCCGCTAAAAGAGCATAG
